# A biofidelic 3D culture model to study the development of brain cellular systems

**DOI:** 10.1038/srep24953

**Published:** 2016-04-26

**Authors:** M. Ren, C. Du, E. Herrero Acero, M. D. Tang-Schomer, N. Özkucur

**Affiliations:** 1 Tufts University, Department of Biomedical Engineering, 4 Colby St., Medford, Massachusetts 02155, USA; 2 Austrian Centre of Industrial Biotechnology Konrad Lorenz Strasse 20, A-3430, Tulln, Austria; 3 Department of Pediatrics, Connecticut Children’s Medical Center & UConn Health, 263 Farmington Avenue, Farmington, Connecticut 06030, USA; 4 Department of Biology, Tufts University, 200 Boston Avenue, Suite 4600, Medford, Massachusetts 02155, USA

## Abstract

Little is known about how cells assemble as systems during corticogenesis to generate collective functions. We built a neurobiology platform that consists of fetal rat cerebral cortical cells grown within 3D silk scaffolds (SF). Ivermectin (Ivm), a glycine receptor (GLR) agonist, was used to modulate cell resting membrane potential (V_mem_) according to methods described in a previous work that implicated Ivm in the arrangement and connectivity of cortical cell assemblies. The cells developed into distinct populations of neuroglial stem/progenitor cells, mature neurons or epithelial-mesenchymal cells. Importantly, the synchronized electrical activity in the newly developed cortical assemblies could be recorded as local field potential (LFP) measurements. This study therefore describes the first example of the development of a biologically relevant cortical plate assembly outside of the body. This model provides i) a preclinical basis for engineering cerebral cortex tissue autografts and ii) a biofidelic 3D culture model for investigating biologically relevant processes during the functional development of cerebral cortical cellular systems.

The selection of experimental models with which to study the biology of development and disease requires researchers to search for components that are specifically targeted to the organism and characteristic of the disease. Some insight into conserved cell biological functions has been provided by 2D tissue cultures, including spheroid cultures grown in a 2D environment, organ-on-chip microfluidic/multi electrode array technologies and cells (cell lines, induced or modified cells) grown in fabricated 3D SFs. The latter of these three are typically referred to as 3D tissue culture models, and they can add more complex cell biological and anatomical relevance to a study^1^,^2^,^3^,^4^. Therefore, these are the critical platforms that are currently available for studying fundamental cellular structures and processes (e.g., synapses and behaviors, growth, differentiation or migration) in response to gene expression/interactions, external stimuli or toxicity. However, when an experimental model is designed for biological and preclinical relevance, it is necessary to noninvasively introduce and maintain the multi-faceted characteristics of a given tissue or organ system for a critical length of time. These systems therefore qualify as alternatives to animal models because cellular-level interactions are imitated in an anatomical and physiological manner as closely as possible to those observed in human biology and disease.

The biofidelic 3D model described in this paper presents a unique design and arrangement of biological, biomaterial and environmental components that can be used to nurture functional self-assembly and maintain the intrinsic functions of brain cellular systems in long term cultures. The purpose of this model is to provide an *in vivo*-like environment to investigate the behavior of brain cellular systems outside of the body while maintaining the highest possible relevance to the system’s natural environment. The model consists of cells that are freshly isolated from rat brain tissue. The model not only keeps the cells alive and functional but it also demonstrates the fundamental biological/electrical/anatomical features of a developing brain at a multicellular systems level over a long period of time. Rat brain cells were chosen because of their high degree of similarity to human brain cells^5^,^6^. Silk biomaterial was chosen for its compatibility with and versatile uses in neurobiological studies^7^.

We evaluated biological parameters that are common across mammalian cellular systems in E18 fetal rat cerebral cortical cells that were grown for up to 3 wks in SF, and spontaneous cortical spike peaks were recorded after 11 wks. Mitochondrial activity, cell population-specific markers, cell resting membrane potentials, action potentials, and mitochondrial transmembrane potentials (ΔΨ_m_) were monitored in three different culture systems: neurons (N), astrocytes (A), and co-cultures (NA). All three of the cellular systems grown in silk-based SFs showed long-term viability. The cells exhibited three distinct distribution patterns: some cells grouped as spherical free aggregates, others as laminar connected aggregates and some grouped as dispersed single cells. These results confirm the anatomical heterogeneity of cerebral cortex cellular systems^8^,^9^,^10^.

Mitochondria, as the drivers of cellular metabolism, play dynamic roles in neuronal differentiation (Supplementary Figure S1) and activity^11^. They have been shown to be altered in several normal and disease states, including diabetes^12^, cancer^13^ and lactating mammary glands^14^. Therefore, analyzing mitochondrial functions and features is a critical step toward assessing cell development, differentiation and functions. Our study describes the changes in mitochondria shape and ΔΨ_m_ that occur during differentiation in rat E18 cerebral cortical cellular systems (Supplementary Data S2). Recent studies have provided extensive evidence indicating that V_mem_ is a useful tool in developmental biology and biomedical studies^15^. V_mem_ has been used as a physiological starting point for examining changes during the stages of cellular proliferation and differentiation^16^ and it is required for persistent directional cell migration^17^. Both of these are fundamental biological processes during tissue development. Neurons in the developing mammalian brain show a Cl^−^-dependent change in their V_mem_ that gradually shifts towards depolarization. A comprehensive description of chloride ion flow-dependent mechanism involved in this V_mem_-shift was first presented by David F. Owens^18^ and later by Ben-Ari and colleagues^19^. Ivm (1 μM) is a GLR-specific agonist^20^ that is not adsorbed on silk materials. This quality ensures that the drug-cell interactions of the drug can be reliably assessed. We used Ivm (1 μM) to specifically control V_mem_ via GLR in the context of neuronal differentiation and growth (Supplementary Figure S3). GLR is the most abundant chloride channel in the central nervous system. Ivm can also modulate GABA-activated Cl channels at ≤0.1 μM, modulate purinergic P2X4 receptors at 3 μM and enhance currents via the α7 neuronal nicotinic acetylcholine receptor at 30 μM. However, in contrast to its specific activity on GLR, Ivm does not display agonist activity at the other mentioned receptors^20^.

## Results

### E18 fetal rat cerebral cortical cells were homogenously distributed and showed different forms of proliferative capacity within the SF environment

**Figure 1:**
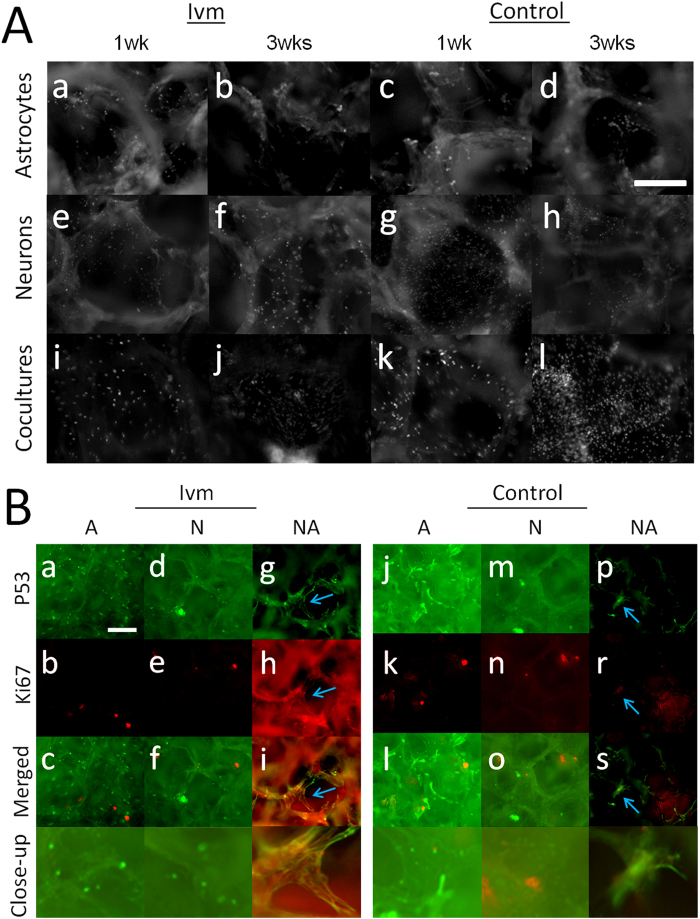
Single cell distribution and proliferation of E18 rat cerebral cortical cell populations grown in 3D SF for 3 wks with or without V_mem_ mediation (Ivm). (**A**) DAPI-stained astrocytes (a–d), neurons (e–h) and co-cultures (i–l) at 1 wk and 3 wks. Each micrograph represents one single pore within a 3D SF. Bar = 0.25 mm. N = 5 SF. (**B**) Ki67- and p53-labeled cell groups that have retained their proliferative capacity and displayed a normal cell cycle at 3 wks. Astrocytes (a–c,j–l), neurons (d–f,m–o) and co-cultures (g–i,p–s) are shown as single or merged views to demonstrate Ki67/p53 double staining. The blue arrows indicate colocalization between Ki67 and p53. N = 6 from 3 SF. Bar = 0.5 mm.

E18 primary rat cerebral cortex cells grown in 3D SF demonstrated three distinct distribution patterns. Some cell groups emerged as spherical buds, some as laminar aggregates, and some were dispersed as single cells. Only the single cells are presented in this section because the other two groups are explained in the following sections. The micrographs in Fig. 1A show nuclei that were stained with DAPI in single cells residing in 3D SF under different culture settings (A, N or NA) and time points (1 wk or 3 wks), with or without V_mem_ modulation (Ivm). Each presented with a different density. Cells were homogenously distributed within or along the walls of the 3D SF cultures (Fig. 1Aa–l). The astrocyte cultures had a similar density regardless of the culture duration or whether Ivm treatment was applied (Fig. 1Aa–d). The neuron cultures and co-cultures were the densest of all of the culture types at 1 wk and 3 wks, respectively (Fig. 1Ag,l). To determine whether rat embryonic cortical cells retained their proliferative capacity in 3D SF, dual immunofluorescence staining for p53 and Ki67, common markers of cell cycle regulation and cell proliferation, respectively, was performed. The fluorescence micrographs in Fig. 1B show cells labeled with p53 and Ki67 in astrocyte cultures, neuron cultures and co-cultures, both with and without Ivm treatment, at 3 wks. At 3 wks, in both the astrocyte (Fig. 1Ba–c,j–l) and the neuron (Fig. 1Bd–f,m–o) cultures, two separate groups of cells were positive for either p53 or Ki67, independent of whether Ivm treatment was applied. On the contrary, cells in co-cultures showed colocalization between p53 and Ki67 under the same conditions. Furthermore, in the co-cultures, largely expanded areas of individual cells revealed an overall cellular distribution instead of a nuclear distribution of p53 and Ki67 (Fig. 1Bg–i,p–s).

### Population-specific changes in mitochondrial activity in E18 fetal rat cerebral cortical cells cultured in SF

**Figure 2:**
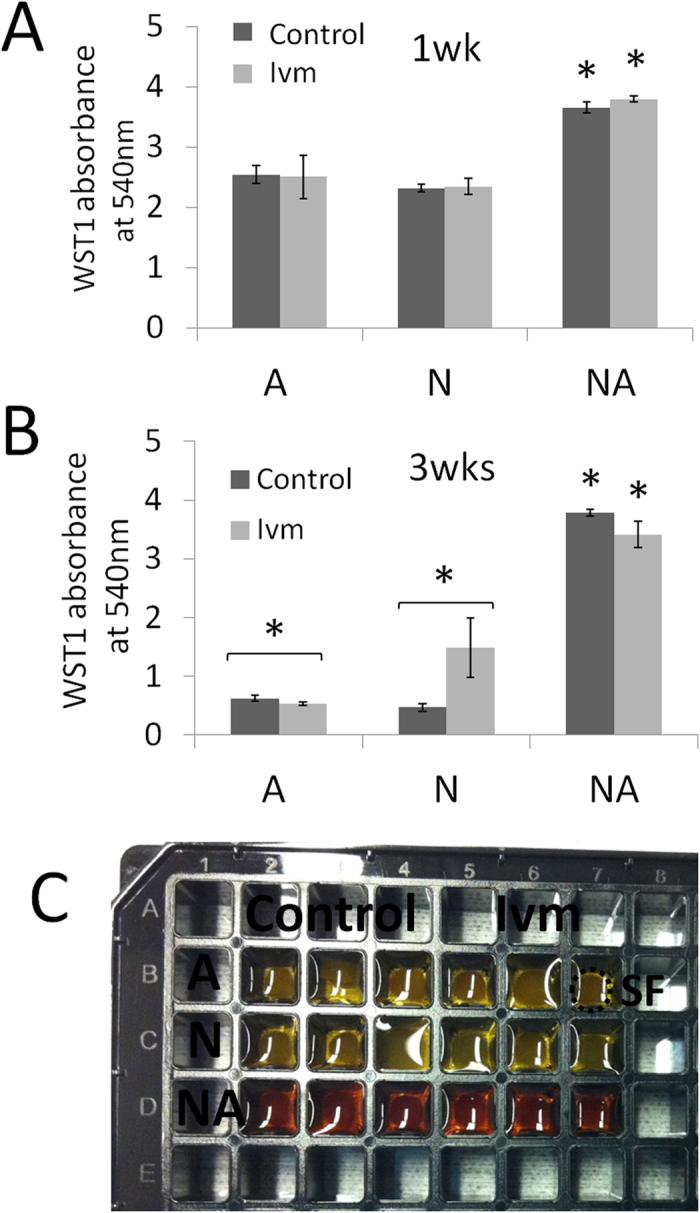
Mitochondrial activity in E18 rat cerebral cortical cell populations that were grown in 3D SF for 3 wks with or without V_mem_ mediation (Ivm). The graphics show the optical density values of WST1 at 540 nm absorbance after 1 wk (**A**) and 3 wks (**B**) in culture. (**C**) A 96-well plate containing cellularized SF shows the visual difference in cell viability and proliferation using mitochondrial activity as an indicator between different cell populations after 2 h of WST-1 degradation. For each condition, data were collected from six separate 3D cultures from 10–15 embryonic cerebral hemispheres. ^*^P ≤ 0.05 versus the other culture types at 1 wk (paired t-test), N = 6 SF.

In addition to acting as an indicator of cell viability, mitochondrial activity has been reported to control the proliferation and differentiation of embryonic stem cells^21^. At 1 wk, the astrocyte and neuron cultures showed similar levels of mitochondrial activity (water soluble tetrazolium salt, WST1, absorbance at 540 nm) both with and without Ivm treatment (Fig. 2A). In the astrocyte cultures, at 3 wks, this activity was reduced 5-fold in both the control and the Ivm-treated cultures. However, at 3 wks, in the neuron cultures, activity was significantly lower, by 5-fold and 1.7-fold, in the control and Ivm-treated cultures, respectively, than in the neuron cultures at 1 wk (Fig. 2B). In contrast, co-cultures showed a significantly higher level of mitochondrial activity than the neuron or astrocyte cultures at both 1 wk and 3 wks, and there was no difference between the cells that were treated with Ivm and the control cells (Fig. 2A,B). The differences in mitochondrial activity across the different cell culture conditions were also visible because the formazan content was the highest (having an orange-red color) in the co-cultures, as shown in Fig. 2C, which shows a snapshot that was taken of a 3 wk SF culture plate.

### Developmental electrophysiology of E18 fetal rat cerebral cortical cells in SF

**Figure 3:**
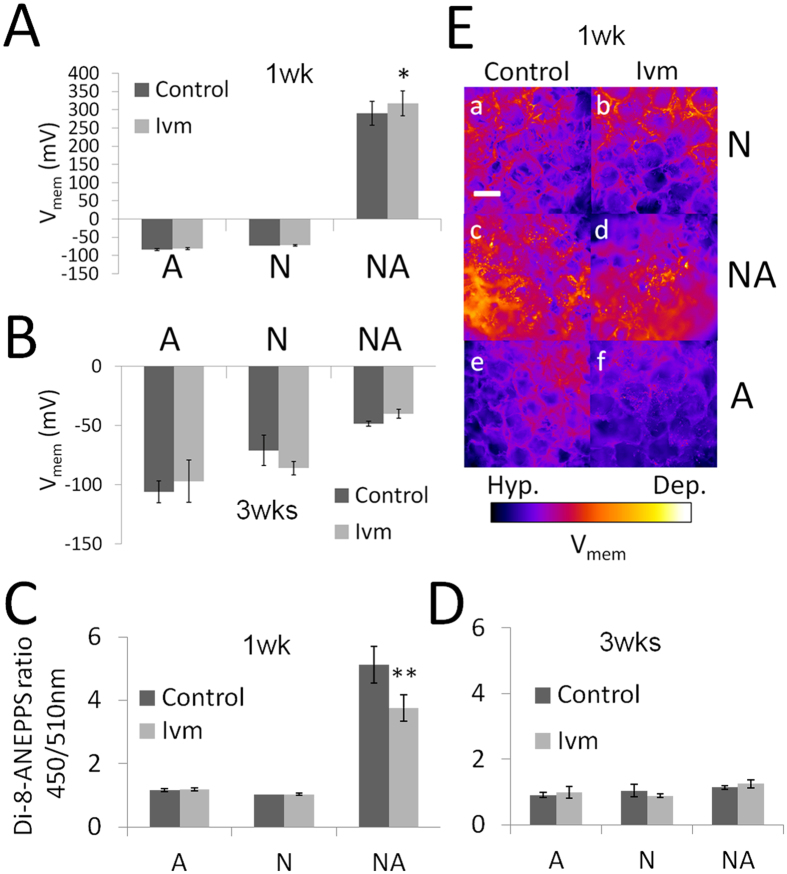
Differences in V_mem_ in E18 rat cerebral cortical cell populations that were grown in 3D SF for 3 wks with or without V_mem_ mediation (Ivm). V_mem_ (mV) values for the astrocyte, neuron and co-cultures at 1 wk (**A**) and 3 wks (**B**) were based on the conversion of di-8ANEPPS ratios that were measured at the same time points (**C**,**D**). (**E**) Examples of optical differentiation of the V_mem_ levels are shown using the di-8-ANEPPS stain in 3D neuron cultures (a,b), cocultures (c,d) and astrocyte cultures (e,f). For each condition, data were collected from three separate 3D cultures that originated from 5–6 embryonic cerebral hemispheres. ^*^P ≤ 0.05 versus A and N, both the control and Ivm, and the NA control, ^*^^*^P ≤ 0.01 versus A and N, both the control and Ivm, and the NA control (paired t-test), N = 3 SF, Bar = 0.5 mm.

Real-time measurements performed using cortical cultures after 1 wk and 3 wks revealed that the cultures displayed long-term changes in V_mem_ that were related to their evolution in the SF environment during the culture period. At a cellular level, long-term changes in V_mem_ have been implicated in cellular differentiation^16^,^22^ (depolarization in neuronal and hyperpolarization in non-neuronal cultures), proliferation^23^ (depolarization in tumor cells) and cancer^24^. Astrocyte cultures displayed an average V_mem_ of −83 mV at 1 wk (Fig. 3A) and at 3 wks were hyperpolarized by −22 mV in the control cultures and −16 mV in the cells exposed to Ivm (Fig. 3B). The neuron cultures showed an average V_mem_ of −72 mV under control conditions at both 1 wk and 3 wks (Fig. 3A,B), whereas they displayed a hyperpolarized V_mem_ of −14 mV at 3 wks when cultured with Ivm (Fig. 3B). Co-cultures had a V_mem_ of +290 mV and +317 mV at 1 wk in the control and Ivm conditions, respectively (Fig. 3A), and a more depolarized V_mem_ than the neurons and astrocytes grown under the same conditions (Fig. 3C). At 3 wks, co-cultures grown under both control and Ivm conditions revealed an average V_mem_ of −45 mV (Fig. 3A), indicating that the cells were more hyperpolarized than they were at 1 wk (Fig. 3C) and more depolarized than the neuron and astrocyte cultures at 3 wks (Fig. 3D). Figure 3E shows false-colored fluorescent micrographs of neurons, astrocytes and co-cultures that were stained with the V_mem_-reporter dye 4-[2-[6-(Dioctylamino)-2-naphthalenyl]ethenyl]-1-(3-sulfopropyl)-pyridinium (di-8-ANEPPS) after 1 wk. The phenotypic relevance of these electrophysiological changes is demonstrated in the following sections. Data reflecting the immediate changes observed in V_mem_ in response to the applied Ivm are shown in the Supplementary Figure S4.

### Tumor screening of cerebral cortical cell cultures as a step towards clinically relevant characterization

**Figure 4:**
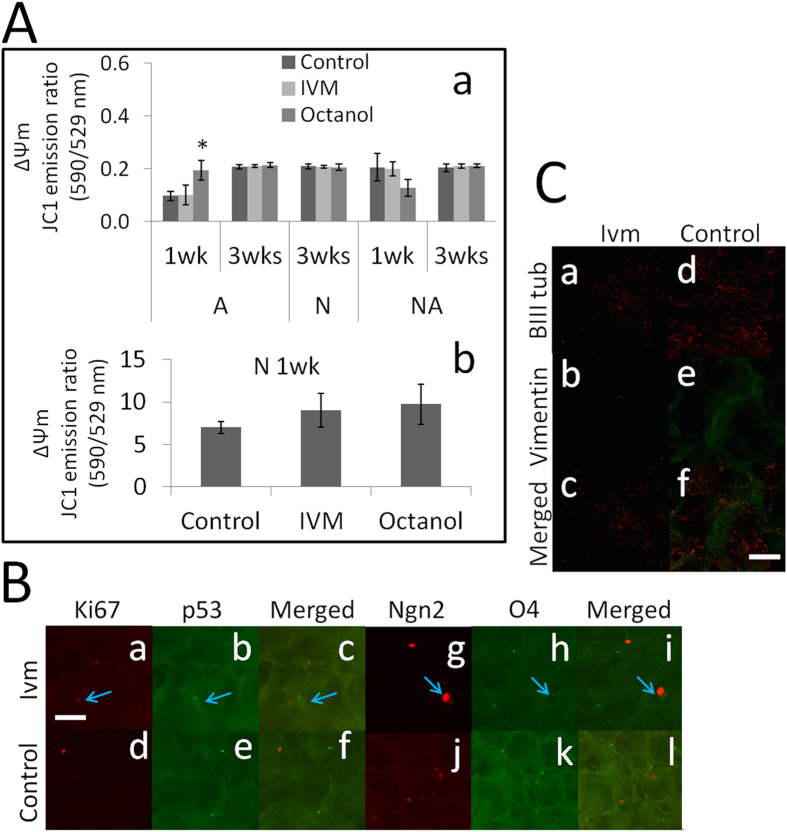
*In vitro* tumor screening of E18 rat cerebral cortical cellular systems was performed using a combined physiological and biochemical assay. (**A**) Fluorescence-based measurements of ΔΨ_m_ levels (as the JC1 dye ratio) were obtained from E18 rat cerebral cortical cell populations that were grown in 3D SF for 3 wks with or without V_mem_ mediation (Ivm) (a,b). An increased JC1 ratio indicated hyperpolarization (tumor feature), as was observed in the neuron cultures after 1 wk, while a decrease indicated depolarization. For each condition, data were collected from three separate 3D cultures that originated from 5–6 embryonic cerebral hemispheres. ^*^P ≤ 0.01 (paired t-test), N = 3 SF. (**B**) Immunocytochemical detection of neoplasm formation in E18 rat cerebral cortical neurons grown in 3D SF for 1 wk with (a–i) and without (d–l) V_mem_ mediation (Ivm). The following antibodies were used: Ki67 (a,d) for proliferative capacity, p53 (b,e) for cell cycle regulation, Ngn2 (g,j) for neural differentiation and O4 (h,k) for oligodendrocyte differentiation. These markers were used to assess normal differentiation versus neoplastic differentiation. The blue arrows in a-c show that separate cell groups were labeled with Ki67 or p53 without colocalization. The blue arrows in g-I show a group of cells that was labeled with Ngn2 without colocalization with O4. Bar = 0.5 mm. N = 6 from 3 SF. (**C**) Immunocytochemical assessment of the epithelial-mesenchymal transition in E18 rat cerebral cortical neurons grown in 3D SF for 1 wk with (a–c) and without (d–f) V_mem_ mediation (Ivm). (a,d) Beta-III tubulin-labeled cells of neuroepithelial origin. (b,e) No cells were labeled with vimentin, which indicates a mesenchymal origin. Bar = 0.25 mm. N = 6 from 3 SF.

*C*ultures were assessed to determine changes in ΔΨ_m_, which is a hallmark of tumor cells. Although the neuronal cultures had a significantly more hyperpolarized ΔΨ_m_ at 1 wk than all of the other cells and conditions, the overall ΔΨ_m_ of the cells was similar at 3 wks (Fig. 4Aa,b). Gap junctions have been shown to elicit mitochondrial depolarization in both astrocytes and neurons grown in monolayer cultures and to provide protection against N-methyl-D-aspartate-induced cytotoxicity^25^. Unlike the V_mem_ (Ivm)-mediated regulation of ΔΨ_m_ via gap junctions that has been observed in 2D cell cultures, cells grown under the same conditions in 3D SF did not display changes in ΔΨ_m_ when they were exposed to Ivm. In addition, ΔΨ_m_ was not affected by the gap junctional blocker Octanol, whereas under similar conditions, treatment with Ivm resulted in significant changes in ΔΨ_m_ in 2D cultures (Supplementary Data S2). This supports the notion that ΔΨ_m_ levels are regulated independent of gap junctions and V_mem_. The only exception was that at 1 wk, the ΔΨ_m_ of Ivm-treated astrocyte cultures were significantly more hyperpolarized when cultured in the presence of Octanol, which is a gap junctional blocker (Fig. 4Aa). An immunocytochemical characterization was then performed to determine whether the transient ΔΨ_m_ hyperpolarization that was observed in neuron cultures (Fig. 4Ab) was associated with tumor markers and whether this might enable the evaluation of neoplasm formation and its origins. Three conditions were assessed: cells were labeled with Ki67 and p53 to assess proliferation and cell cycle regulation, respectively; cells were labeled with Ngn2 in conjunction with O4 to assess their neuronal origin; and cells were labeled with vimentin in conjunction with Beta-III tubulin to assess their epithelial-mesenchymal origin^26^. At 1 wk, the neuron cultures formed spherically aggregated cell groups that expressed either Ki67 or p53 or spheres that were positive for either Ngn2 (postmitotic neurons) or O4 (immature oligodendrocytes). Both of these markers indicate that the cells had a neural origin (Fig. 4B). At 1 wk, the neuron cultures contained single cells that expressed Beta-III-tubulin but not vimentin (Fig. 4C). These data indicated that at 1 wk, the cells in the neuron cultures had an epithelial origin but were not mesenchymal.

### E18 rat cerebral cortical cells form physically and phenotypically distinct aggregates after 3 wks in an SF environment

#### Neuron cultures

**Figure 5:**
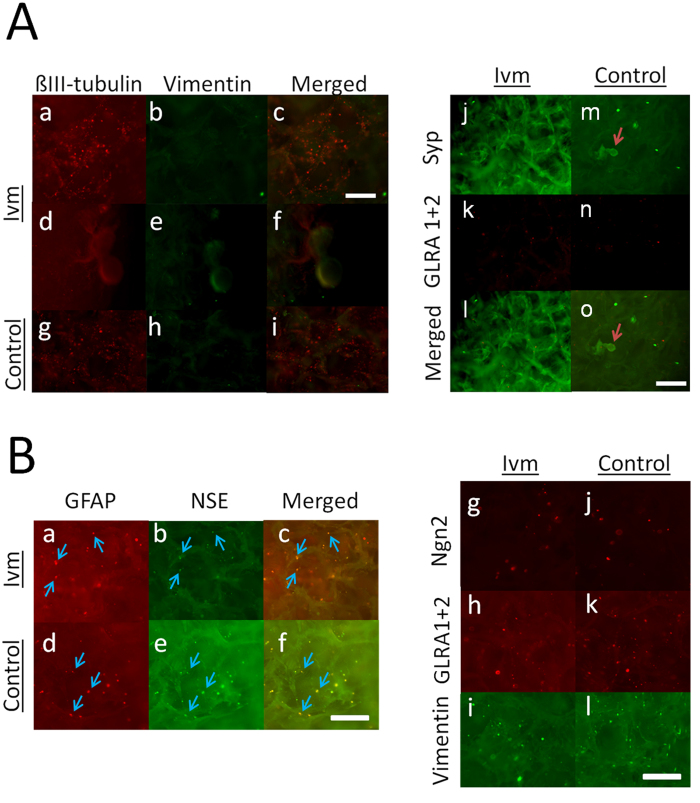
Physically and phenotypically distinct cell aggregates were observed in E18 rat cerebral cortical cells that were grown in homotypic cultures for 3 wks in 3D SF. (**A**) Immunocytochemical characterization of the epithelial-mesenchymal transition in neuron cultures grown with or without V_mem_ mediation (Ivm). The Ivm-treated neuron cultures showed cell groups that stained with Beta-III tubulin, but no cells were labeled with vimentin (a–c). Spherical buds consisted of cells that showed co-localized Beta-III tubulin/vimentin labeling (d–f). The control cultures showed some cell groups that were stained with Beta-III tubulin and a small number of cell groups that stained with vimentin (g–i). The Ivm-treated neuron cultures showed cell groups that were stained with the synaptic proteins Syp or GLRA1 + 2 (j–l). The control cultures showed spherical buds (arrows) that consisted of cells displaying Syp/GLRA1 + 2 colocalization and a few single cells that were labeled with GLRA1 + 2 or small aggregates of cells that were labeled with Syp (m–o). N = 6 from 3 SF. (**B**) Immunocytochemical characterization of cell groups in astrocyte cultures grown with or without V_mem_ mediation (Ivm). In both the Ivm and the control conditions, the astrocyte cultures showed small spherical aggregates that were approximately 50 μm in size that consisted of cells with GFAP/NSE colocalization, indicating a neuroglial origin (a–f). Bars = 0.5 mm. N = 6 from 3 SF.

E18 fetal rat cerebral cortical cells that were grown in SF displayed distinct distribution patterns when grown under different conditions. Neuron cultures showed an intense and homogenously distributed group of Beta-III tubulin-labeled cells that contained no vimentin labeling under both Ivm-treated (Fig. 5a–c) and non-treated conditions (Fig. 5Ag–i). In addition, neurons formed spherical buds consisting of both Beta-III tubulin- and vimentin-labeled cells under the Ivm-treated condition (Fig. 5Ad–f). Neuron cultures were stained for the synaptic proteins Synaptophysin (Syp) and GLRA1 + 2 to detect the recruitment of pre and postsynaptic proteins, respectively. Treatment with Ivm resulted in a singular distribution of cells with an elongated morphology (Syp) that displayed an overall distribution with a dot-like staining pattern (GLRA1 + 2) (Fig. 5Aj–l), while sphere-shaped aggregates that consisted of both Syp- and GLRA1 + 2-labeled cells were observed in the cells grown under control conditions (Fig. 5Am–o).

#### Astrocyte cultures

Except for the p53-labeled cell group that was described in the previous section, the cells in the primary astrocyte cultures showed a round-shaped morphology (Fig. 5B, blue arrows). Cells in the primary astrocyte cultures also showed a less intense distribution pattern within SF than the densely packed B-III tubulin-producing cells observed in the neuron cultures, potentially indicating a migratory state. Cells in primary astrocyte cultures also showed co-localization between NSE and GFAP (Fig. 5Ba–f) and cell groups that contained cells that produced Ngn2, GLRA1 + 2 or vimentin (Fig. 5Bg–l). These data potentially indicate the presence of neuroglial stem/progenitor (NSE and GFAP), postmitotic (Ngn2), postsynaptic (GLRA1 + 2) and epithelial-mesenchymal (vimentin) features, respectively. The plasticity of the cells at this stage of brain development prevented us from concluding that any one marker was indicative of one particular cell type. We therefore assessed the different populations of rat E18 cerebral cortical cells with respect to their developmental stages in the light of the findings of previous studies. For example, vimentin has been shown to be a marker for astrocytes that are transitioning from an immature to a mature state, and vimentin is expressed before GFAP during brain development^27^. Vimentin expression is also characteristic of neuroepithelial cells and radial glial cells (neural stem/progenitors)^28^, and radial glia also express GFAP. GFAP is therefore another marker of postnatal neural precursors in the developing brain^29^. In addition, NSE can be used to assess neuroglial progenitor cells because its expression is not unique to neural cells in the developing CNS^30^.

#### Co-cultures

**Figure 6:**
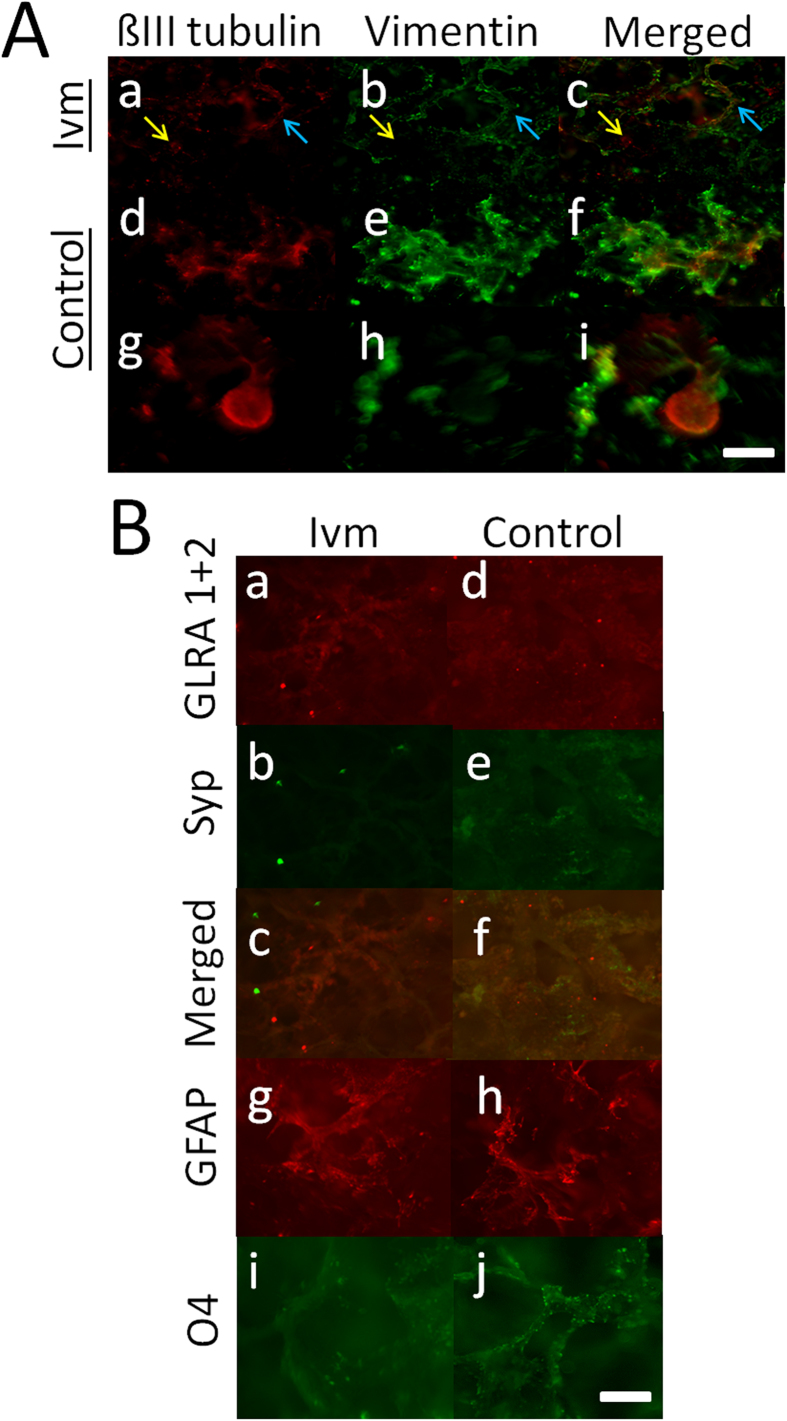
Physically and phenotypically distinct cortical cell aggregates were observed in co-cultures of neurons and astrocytes that were grown for 3 wks in 3D SF. (**A**) Immunocytochemical characterization of the epithelial-mesenchymal transition in co-cultures grown with or without V_mem_ mediation (Ivm). The Ivm-treated co-cultures showed single cell groups that displayed Beta-III tubulin/vimentin colocalization (blue arrow) and separate groups of cells that were labeled with either Beta-III tubulin or vimentin (yellow arrow) (a–c). The control co-cultures showed patches (d–f) or spherical buds that were approximately 250 μm in size (g–i), and both groups consisted of cells that were labeled with Beta-III tubulin and/or vimentin. N = 6 from 3 SF. (**B**) Immunocytochemical characterization of cells expressing synaptic proteins (Syp and GLRA1 + 2), GFAP-producing astrocytes and oligodendrocytes (O4) that were grown in co-cultures with or without V_mem_ mediation (Ivm). The Ivm-treated co-cultures show densely packed cell groups that were labeled with GLRA1 + 2 (no Syp) and small spherical aggregates that were 10–40 μm in size that were labeled with GLRA1 + 2 or Syp (a–c). The control co-cultures showed densely packed cells that were labeled with GLRA1 + 2 and/or Syp and small, spherical aggregates that were labeled with only GLRA1 + 2 (no Syp) (d–f). Both the Ivm-treated and the control co-cultures showed densely packed but separate groups of cell that were labeled with either GFAP or O4. N = 6 from 3 SF. Bars = 0.25 mm.

Co-cultures showed singular distributed cells that expressed Beta-III tubulin or vimentin, and partial colocalization was observed between the two in the Ivm-treated cells (Fig. 6Aa–c). The control cells formed either irregularly shaped patches (Fig. 6Ad–f) or spherical buds (Fig. 6Ag–i), and both were labeled with Beta-III tubulin and/or vimentin in a manner similar to that observed in the Ivm-treated cells. Co-cultures also contained GLRA1 + 2- and Syp-expressing cells, singular distributed cell groups that expressed GLRA1 + 2, and spherical buds that expressed GLRA1 + 2 or Syp when grown in the presence of Ivm (Fig. 6Ba–c). The control plates contained singular distributed cell groups that were labeled with both GLRA1 + 2 and Syp (Fig. 6Bd–f) in addition to separate groups of GFAP- (Fig. 6Bg,h) or O4-expressing cells (Fig. 6Bi,j), with the latter growing along the edges of the SF pores.

### V_mem_ as a physiological switch between neural stem/progenitor cell niche retention and cortical neuron network development

**Figure 7:**
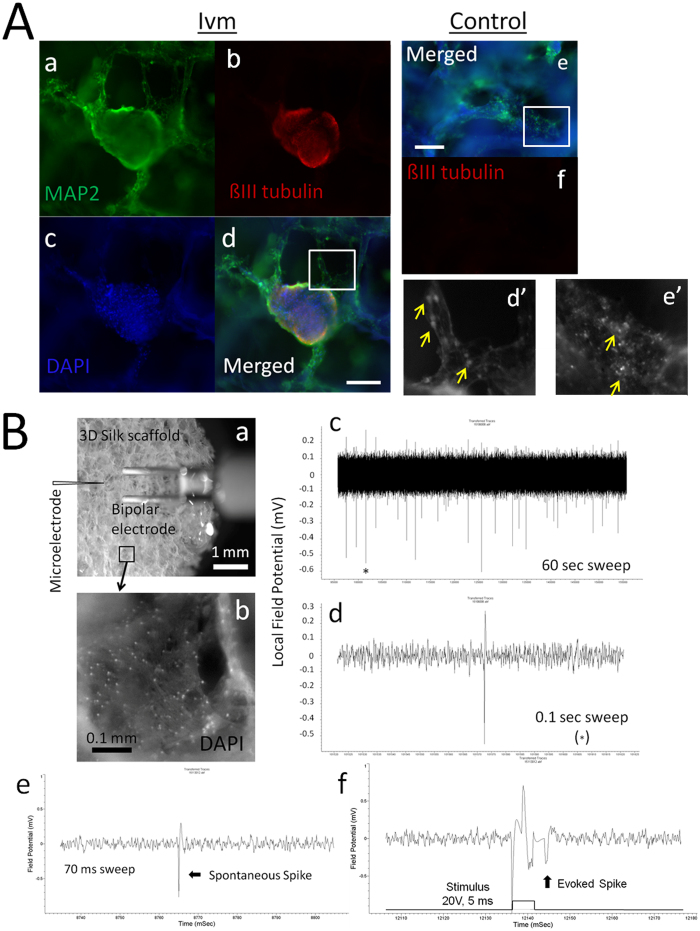
A V_mem_ switch was observed between long-term neural stem/progenitor cell niche retention and functional assembly of the cortical plate. (**A**) Samples of E18 rat embryonic cortical tissues were grown in 3D SF for 11 wks with (a–d,d’) or without V_mem_ mediation (Ivm) (e,f,f’). (a–d) Spherical buds consisting of Beta-III tubulin-expressing inner core cells and MAP2-expressing outer layer cells generated corridors of neurons that extended towards the tissue layer growing below. DAPI was used to label cell nuclei. (d’) A single corridor of MAP2-labeled neurons (yellow arrows) bridging the spherical bud and the underlying tissue. (e,f) The connective cortical neurons were labeled with MAP2 but not with Beta-III tubulin in the control cultures. (e’) Cortical neurons labeled with MAP2 (yellow arrows) showed a laminar organization. Bar = 0.2 mm. N = 6 from 3 SF. (**B**) EFP recordings from E18 rat embryonic cortical tissues that were grown in 3D SF for 11 wks. (a) Brightfield image showing the EFP set-up within the petri dish: a bipolar electrode and a microelectrode were placed near the SF. Bar = 1 mm. (b) A close-up view of a pore filled with a laminar layer of cells that were labeled with DAPI. Bar = 0.1 mm. (c,d) Spontaneous spikes are shown in a 60 sec sweep or an expanded 0.1 sec sweep in which a single spike (*) was recorded from E18 rat embryonic cortical tissue grown in 3D SF for 11 wks without V_mem_ mediation. (e) A spontaneous spike is shown in a 70 ms sweep, and (f) a stimulus-evoked spike (20 mV, 5 ms) is shown that was recorded from a single group of cells. N = 3 SF.

Long-term (11 wks) cultures of E18 fetal rat cerebral cortical neurons grown in SF in the presence of Ivm contained spheres that were 400 μm in diameter. These spheres consisted of Beta-III tubulin- and MAP2-labeled cells that grew in an inside-out fashion (Fig. 7Aa–d) and were physically connected to the surrounding cell groups (MAP2) via 4–5 corridors of migrating cells (Fig. 7Ad,d’). At 11 wks, the cortical neuron cultures grown under control conditions contained a homogenous distribution of MAP-producing cells but no Beta-III tubulin-labeled cells or spheres (Fig. 7Ae–f). At 11 wks, cortical neurons grown under control conditions exhibited a more densely packed laminar assembly of MAP2-producing cells than the neurons grown in the presence of Ivm (Fig. 7Ae,e’). Both the control and the Ivm-treated cortical neuron culture systems produced real-time, synchronized and spontaneous evoked electrical spikes, which were measured during EFP recordings. The EFP recordings obtained from the control culture neurons at 11wks are shown in Fig. 7Ba,b. The neuronal activities of the neurons grown in 3D SF are presented as a 60-sec sweep (Fig. 7c) and as a 0.1-sec sweep (Fig. 7d). Spontaneous and stimulus-evoked spikes are shown in detail (Fig. 7Be,f). Spikes were defined as sharp changes in the local field potential in the sub millisecond range and were counted as events when they crossed the detection threshold, which was set at a time corresponding to the average peak-to-peak amplitude of baseline noise. The baseline noise measurements of the recordings were between 0.25 and 0.3 mV. Spike frequencies ranged from ≤0.016 Hz up to 0.13 Hz, and one single measurement at 0.47 Hz was collected from the E18 rat cerebral cortical aggregates grown in 3D SF during 3 months of culture under different conditions. The amplitudes of the events displayed a continuous scale from the detection threshold (0.25–0.3 mV) to the data acquisition’s max amplitude of 1 mV because of we used an amplification factor of x10,000. Cultures were maintained for up to 6 months or until they had to be discarded as a result of contamination.

## Discussion

The data obtained in this study suggest that long-term co-cultures grown in SF might reflect an *in vivo*-like, gradual state of development between stemness and terminal differentiation because they displayed a more depolarized V_mem_ at 3 wks than at 1 wk (neuronal differentiation^31^). No change was observed in mitochondrial activity (stemness retained^32^) or in the depolarized ΔΨ_m_, contrary to the hyperpolarized ΔΨ_m_ that is typically observed in tumor cells^33^. This indicated that proliferation and differentiation were ongoing processed in the co-cultures.

Unlike the direct relationship that was revealed in the 2D studies (Supplementary Data S2), the changes in ΔΨ_m_ in the 3D cultures grown in SF were independent of gap junctions between embryonic cerebral cortical cells. This implies that these cells can be used as a model of developing cortex. These results are similar to results showing that these junctions disappear in the developing cortex, possibly because of the transient disappearance of the gap junction-mediated coupling of neurons or of the gap junctions themselves^34^. Otherwise, it is possible that an unknown regulator of ΔΨ_m_ could be responsible at that specific stage of development.

The transient depolarizations exceeding 300 mV that were observed in the 1 wk co-cultures may have simply reflected the progression of the 3D culture system. In combination with the observation that changes in resting potential were on the order of 100 mVs, these differences were significantly independent of intrinsic V_mem_ shifts, which occur on the order of 10 mVs during neural development^35^,^36^, and of changes that occur in response to temperature differences, which are on the order of 10%^37^.

Another result of this study might be related to a recently emergent and intriguing phenomena: the potential of a link between brain development and breast cancer^38^. Some cell groups in the co-cultures grown in SF exhibited colocalization between and an overall cytoplasmic/membrane distribution for p53 and Ki67. This is a phenotype that has only been reported in breast cancer cells^39^,^40^. Hence, it might be important to expand further studies to better understand the origin and importance of this link.

Recent research has shown that embryonic stem cells and cancer stem cells not only share common biological features but also experience similar conditions in their microenvironment, such as an acidic pH^41^, that are different from those experienced by lineage-committed cells^17^. Therefore, tuning cellular biology alone may not be sufficient to rule out neoplastic tissue formation. Instead of intervening in the biology of the cell using genetic methods, a multi-faceted approach that also takes into account the extracellular (or non-cellular) parameters might provide cells with a natural environment in which they can grow and progress without invasive manipulations. The divergent mechanisms that cause neoplasm formation could be determined by studying normal tissue formation. Our data have implicated ΔΨ_m_ as a differential characteristic between stemness and tumorigenicity in cells grown *in vitro* in 2D cultures of dissociated cortical cells because the percentage of p53-positive cells decreased despite the fact that the number of proliferating (Ki67-positive) cells increased after 3 wks of treatment with Ivm (Supplementary Data S2 and Supplementary Figure S5). Thus, ΔΨ_m_ was used as a diagnostic tool for tumor screening in the cultures grown in SF.

Self-assembled cerebral cortex cells grown in SF showed anatomical structures that are similar to those observed during *in vivo* corticogenesis at the cellular systems level. The neuron cultures showed a laminar assembly of cells (MAP2-labeled dendrites) that resembled a cortical plate and the ability to generate synchronized population spikes. They therefore presented anatomical and physiological similarities to cortical microcircuit assembly^42^,^43^. Neuron cultures grown in the presence of Ivm demonstrated the ability to grow corridors of migrating neurons that originated from the Beta-III tubulin/MAP2-producing neural stem/progenitor cell niche. These corridors of migrating neurons have been observed in the developing human brain during the pre- and early postnatal stages^44^. The bud-like morphology of this niche resembled the forebrain vesicle that is formed at the front end of the neural tube in developing mammalian embryos^45^.

Tissue elasticity can direct stem cell lineage specification^46^, and it has also been found to increase during adult neurogenesis^47^. Cultures grown in 3D SF have been engineered with specific biomechanical properties^7^ that closely match the biomechanical properties of developing mammalian cerebral cortex tissues^48^. The combination of bioengineered SF, 3D cues and the specific modulation of intrinsic physiology (V_mem_) enabled the long-term retention of the neural stem/progenitor niche and a functionally and biologically relevant model of mammalian cerebral cortex development.

Given recent findings that have implicated endogenous V_mem_ in the formation of cerebral cortical assemblies and connectivity *in vitro*^49^, in the migration of neuroblasts from the subventricular zone^50^, and in the proliferation of cells during *Xenopus laevis* neural tissue development^51^, the exploitation of bioelectric pathways is likely to represent an important step that will enable an increased understanding of brain development and the engineering of biologically relevant tissues. The 3D culture model presented in this paper is relevant to early human brain development at a cellular systems level. These systems can therefore serve as a robust tissue model for biological and clinical studies of early brain development at a multicellular systems level. The model also provides a basis for future studies aimed at growing *ex vivo* niches of different populations of neonatal cerebral cortical cells, and our findings may therefore serve as a biologically and biomedically relevant platform for developing novel cellular therapies.

## Materials and Methods

### Animal studies and preparation of rat cortical cells

E18 rat (*Rattus norvegicus*, Sprague-Dawley, Charles River) cerebral cortical tissues were generously provided every week by Dr. Steven Moss’s laboratory at Tufts Neuroscience Center. The brain tissue isolation protocol was approved by the Tufts University Institutional Animal Care and Use Committee and complied with the NIH Guide for the Care and Use of Laboratory Animals (IACUC # B2011–45). The isolation and dissociation of the rat cortical neurons and astrocytes and the preparation of the cell cultures were performed as previously described^49^. Neural media (Neurobasal + B27 Supplement) was specifically developed for enriching neurons. Previous studies have reported that astroglia comprise <0.5% of the cell population in neuronal cultures grown in Neurobasal-B27^52^,^53^. The growth of other types of cells is greatly inhibited as a result of the presence of biophysical parameters that are advantageous for neurons but not for astrocytes. A complete elimination of GFAP-positive cells can be achieved using anti-mitotic agents, such as cytarabine, to inhibit astrocyte proliferation. However, this reduces neuronal cell health because these chemical agents are toxic to neural functions. Therefore, they were not included in the studies presented in this paper.

### Chemicals

Ivermectin was obtained from Tocris Bioscience (Cat# 1260, Bristol, UK). Stocks of 10 mM ivermectin were dissolved in dimethyl sulfoxide and stored at −20 °C. Ivm was used at a final concentration of 1 μM. Stocks of 127 μM octanol (8-OH) were prepared by diluting 10 μl of 1-octanol (Cat# O4500, Sigma-Aldrich) in 500 mL of distilled water. Octanol was used at a final concentration of 2.54 nM. For safety the information for Ivermectin and octanol, please refer to their product websites.

### Silk Scaffold Preparation

A silk solution was prepared from *B. mori* cocoons as previously described^54^. Porous scaffolds were constructed using a salt leaching method as previously described^55^. Briefly, a concentrated silk fibroin solution (6% w/v in water) was impregnated at a volume-to-weight ratio of 1:2 with 500–600 μm-sized sodium chloride particles (Sigma-Aldrich, Natick, MA, USA) that were pre-sorted using test-grade metal sieves. After 48 h, the silk fibroin/salt mixture was immersed in water, and the sodium chloride was dissolved over a couple of days, during which fresh water was exchanged with the water in the solution. The remaining insoluble silk material displayed a large porous silk scaffold. The scaffold was cut into cylindered disks that were 4 mm in diameter and 2 mm in height. The scaffolds were autoclaved in distilled water and stored at 4 °C when not in use.

Ivm adsorption data were obtained during the monitoring of dynamic drug adsorption on the silk films using a quartz crystal microbalance, and these data can be found in the online Supplementary Data S6.

### Seeding of Cells on Silk Scaffolds

The E18 fetal rat cerebral cortical cells were dissociated as previously described^49^. Silk scaffolds were placed in a 24-well plate, and 0.5 mL of a 0.1 mg/mL solution of poly-D-lysine (Sigma-Aldrich, Natick, MA, USA) was added. The scaffolds were allowed to soak for 30 minutes before the solution was aspirated, and they were then washed with distilled water before they were left to air dry. Astrocytes were added directly onto the coated dry scaffolds at a concentration of 1 × 10^5^ cells per scaffold in phenol-free DMEM supplemented with 1% penicillin/streptomycin, 2% GlutaMAX, and 2% FBS (Invitrogen, Carlsbad, CA, USA). Neurons were added at 3 × 10^5^ cells per scaffold to the other scaffolds in phenol-free neurobasal medium supplemented with B27, 1% penicillin/streptomycin, and 1% GlutaMAX. The cells were incubated at 37 °C, and the cell culture media were changed once per week. The same number of cells was used to co-culture the astrocytes and neurons.

### High-throughput V_mem_ measurement

We used ratiometric di-8-ANEPPS dye (D-3167, Invitrogen, Carlsbad, CA, USA) to measure the V_mem_ of neuron cultures (N), astrocyte cultures (A) and cocultures (NA) for 0–4 weeks after plating. The cells were washed once with Hank’s Balanced Salt Solution (HBSS) prior to staining. Di-8-ANEPPS dye was diluted in HBSS to a final concentration of 2 μM and then added to the cells (100 μL/well). The cells were incubated at 37 °C for 30 min in the dark. Dye uptake was confirmed under a microscope using a red filter. The cells were washed once with HBSS to remove excess dye prior to measurement. Fluorescent intensities were measured using a top-read fluorescence multimode multiplate reader (SpectraMax M2, Molecular Devices, Sunnyvale, CA, USA). The relative V_mem_ of the cells was measured as the ratio of readings at two excitation sites of the dye (450 nm/510 nm, emission: 640 nm). The plots were generated using Excel.

### *High-throughput ΔΨ*_*m*_ *measurement and visualization*

The dye 5,5′,6,6′-tetrachloro-1,1′,3,3′-tetraethylbenzimi-dazolylcarbocyanine iodide (JC1) (T3168, Invitrogen, Carlsbad, CA, USA) was used to measure the ΔΨ_m_ of neuron cultures (N), astrocyte cultures (A) and cocultures (NA) at 0–4 wks after plating. The cells were washed once with HBSS prior to staining. The JC1 dye was diluted in HBSS to a final concentration of 5 μM and then added to the cells (100 μL/well). The cells were incubated at 37 °C for 30 min in the dark. The incubation time and the final JC1 concentration used for the primary cultures of rat cortical neurons and astrocytes were chosen based on previous studies^56^,^57^. The cells were washed once with HBSS to remove excess dye prior to measurement. Fluorescence intensities were measured using a top-read fluorescence multimode multiplate reader (SpectraMax M2, Molecular Devices, Sunnyvale, CA, USA) and SoftMax Pro software. The relative ΔΨ_m_ of the cells was measured as the ratio of readings at two emission sites of the dye (red/green: 590 nm/529 nm, excitation: 488 nm). The plots were generated using Excel. To assess the mitochondrial shapes shown in Supplementary Data S2, images were taken of the green JC1 monomers, whereas the JC1 red aggregates indicated the changes in Ψ_m._

### Immunocytochemistry

A basic immunocytochemistry protocol was performed as previously described^49^. The following antibodies were used for the analysis: mouse monoclonal oligodendrocyte marker 4 (1:50, O4, Sigma-Aldrich Cat. # O7139, RRID:AB_477662), mouse monoclonal glial fibrillary acidic protein (1:100, GFAP, Sigma-Aldrich Cat. # G3893, RRID:AB_477010), rabbit polyclonal glycine receptor alpha 2 (1:100, GLRA 2, Abcam Cat. # ab97628, RRID:AB_10680442) rabbit polyclonal glycine receptor alpha 1 + 2 (1:1000, GLRA1 + 2, Abcam Cat. # ab23809, RRID:AB_2110062), rabbit polyclonal neurogenin 2 (1:100, NGN2, Abcam Cat. # ab26190, RRID:AB_2266957), mouse monoclonal synaptophysin (1:25, Abcam Cat. # ab8049, RRID:AB_2198854), mouse monoclonal vimentin (5 μg/mL, Abcam Cat. # ab8069, RRID:AB_306239), rabbit polyclonal beta III-tubulin (1:1000, Abcam Cat. # ab18207, RRID:AB_444319), anti-mouse monoclonal p53 (1:100, Abcam Cat. # ab26, RRID:AB_303198), anti-rabbit Ki67 (1:500, Cat. # ab15580, RRID:AB_443209), anti-mouse monoclonal MAP2 (Abcam, Cat. # ab11267 RRID: AB_297885), anti-mouse Alexa Fluor 568 (1:100, Life Technologies Cat. # A11004 RRID:AB_10562368), anti-rabbit Alexa Fluor 488 (1:100, Molecular Probes Cat. # A11008 RRID:AB_143165), and 4′,6-diamidino-2-phenylindole (1 μg/mL, DAPI, Invitrogen, Carlsbad, CA, USA). Images were collected using 20X and 40X objectives on a Leica DM (Wetzlar, Germany) inverted fluorescence microscope that was equipped with a mercury lamp, an ebq 100 and a monochrome camera. The DAPI (excitation 360 ± 20 nm and emission 470 ± 20 nm), green fluorescent protein (GFP, excitation 470 ± 20 nm and emission 525 ± 25 nm) and Texas Red (excitation 560 ± 20 nm and emission 645 ± 40 nm) filter sets were used.

### Mitochondrial activity assay

Mitochondria-based tetrazolium salt WST-1 assays were used to assess mitochondrial activity according to the manufacturer’s instructions (Roche, 5015944001). Cultures were plated for 0–4 weeks and then incubated for 2 hours at 37 °C with the WST-1 assay reagent (1:10 in cell culture medium). Mitochondrial dehydrogenase activity was measured as a function of mitochondrial activity at 440 nm using a multimode multiplate reader (SpectraMax M2, Molecular Devices, Sunnyvale, CA, USA) with SoftMax Pro software.

### EFP Recording

Dissociated E18 rat embryonic cortical neurons were seeded on poly-D-lysine-coated 35-mm Corning Culture Dishes or in 3D silk protein-based scaffolds. After a period of incubation (10 days to 3 months), the electrical spiking activities of the neurons grown in cultures and on 3D scaffolds were recorded extracellularly at room temperature using sharp glass microelectrodes and an NPI Bridge Amplifier (BA-03X, Tamm, Germany). The cells grown in cultures and on 3D scaffolds were immersed in extracellular solution (140 mM NaCl, 2.8 mM KCl, 2 mM CaCl_2_, 2 mM MgCl_2_, 10 mM HEPES, and 10 mM D-glucose, with pH adjusted to 7.4 using NaOH). Sharp glass microelectrodes were pulled using Sutter Borosilicate Glass (BF150-86-10) with a Sutter Micropipette Puller (P-87). The microelectrodes were filled with extracellular solution and had a resistance of 60–80 Mohm. The cell cultures and 3D scaffolds were visualized using a Wild M3C Dissecting Microscope (Switzerland). The recording electrodes were positioned close to the cultured tissues. Fast local field potential changes (spikes) were recorded using the NPI amplifier at a bandwidth of 0.3–10 kHz and then further amplified using an A-M Systems Differential AC amplifier (Model 1700) to a combined total gain of 10,000×. The signals were digitized at 10 KHz using a Molecular Devices digitizer (DigiData 1550) using a Dell OptiPlex GX620 computer with pClamp 10 software (Molecular Devices). The electrical stimulations of the cultured tissues were generated using a Grass S44 Stimulator, passed through a Grass Stimulus Isolation Unit (SIU5), and delivered using a platinum parallel bipolar electrode (FHC PBSB0875). There was a distance of 800 μm between the two tips, which were positioned near the recording electrode. Images showing the measurement setup, which consisted of scaffolds and electrodes, were captured using an AmScope microscope digital camera (MU1000-CK).

### Statistical analysis

Statistical calculations were performed using paired or unpaired Student’s t tests. Significant was defined as p < 0.05 (*) or p < 0.01 (**). The data are presented as the mean ± SEM.

## Additional Information

**How to cite this article**: Ren, M. *et al*. A biofidelic 3D culture model to study the development of brain cellular systems. *Sci. Rep.* **6**, 24953; doi: 10.1038/srep24953 (2016).

## Supplementary Material

Supplementary Information

## References

[b1] Pasca A. M. . Functional cortical neurons and astrocytes from human pluripotent stem cells in 3D culture. Nat Methods 12, 671–678 (2015). 26005811 10.1038/nmeth.3415 PMC4489980

[b2] Thonhoff J. R., Lou D. I., Jordan P. M., Zhao X. & Wu P. Compatibility of human fetal neural stem cells with hydrogel biomaterials *in vitro*. Brain Res 1187, 42–51 (2008). 18021754 10.1016/j.brainres.2007.10.046 PMC2176077

[b3] van Duinen V., Trietsch S. J., Joore J., Vulto P. & Hankemeier T. Microfluidic 3D cell culture: from tools to tissue models. Curr Opin Biotechnol 35, 118–126 (2015). 26094109 10.1016/j.copbio.2015.05.002

[b4] Bosi S. . From 2D to 3D: novel nanostructured scaffolds to investigate signalling in reconstructed neuronal networks. Sci Rep 5, 9562 (2015). 25910072 10.1038/srep09562 PMC5407555

[b5] Narayanan N. S., Cavanagh J. F., Frank M. J. & Laubach M. Common medial frontal mechanisms of adaptive control in humans and rodents. Nat Neurosci 16, 1888–1895 (2013). 24141310 10.1038/nn.3549 PMC3840072

[b6] Sauvageot C. M. & Stiles C. D. Molecular mechanisms controlling cortical gliogenesis. Curr Opin Neurobiol 12, 244–249 (2002). 12049929 10.1016/s0959-4388(02)00322-7

[b7] Tang-Schomer M. D. . Bioengineered functional brain-like cortical tissue. Proc Natl Acad Sci USA 111, 13811–13816 (2014). 25114234 10.1073/pnas.1324214111 PMC4183301

[b8] Lerch J. P. . Mapping anatomical correlations across cerebral cortex (MACACC) using cortical thickness from MRI. Neuroimage 31, 993–1003 (2006). 16624590 10.1016/j.neuroimage.2006.01.042

[b9] Hoerder-Suabedissen A. & Molnar Z. Development, evolution and pathology of neocortical subplate neurons. Nat Rev Neurosci 16, 133–146 (2015). 25697157 10.1038/nrn3915

[b10] Bystron I., Blakemore C. & Rakic P. Development of the human cerebral cortex: Boulder Committee revisited. Nat Rev Neurosci 9, 110–122 (2008). 18209730 10.1038/nrn2252

[b11] Kim D. Y., Rhee I. & Paik J. Metabolic circuits in neural stem cells. Cell Mol Life Sci 71, 4221–4241 (2014). 25037158 10.1007/s00018-014-1686-0 PMC4394599

[b12] Sivitz W. I. & Yorek M. A. Mitochondrial dysfunction in diabetes: from molecular mechanisms to functional significance and therapeutic opportunities. Antioxid Redox Signal 12, 537–577 (2010). 19650713 10.1089/ars.2009.2531 PMC2824521

[b13] Warburg O. On the origin of cancer cells. Science 123, 309–314 (1956). 13298683 10.1126/science.123.3191.309

[b14] Rosano T. G., Lee S. K. & Jones D. H. Developmental changes in mitochondria during the transition into lactation in the mouse mammary gland. II. Membrane marker enzymes and membrane ultrastructure. J Cell Biol 69, 581–588 (1976). 178667 10.1083/jcb.69.3.581 PMC2109714

[b15] Levin M. & Stevenson C. G. Regulation of cell behavior and tissue patterning by bioelectrical signals: challenges and opportunities for biomedical engineering. Annu Rev Biomed Eng 14, 295–323 (2012). 22809139 10.1146/annurev-bioeng-071811-150114 PMC10472538

[b16] Sundelacruz S., Levin M. & Kaplan D. L. Role of membrane potential in the regulation of cell proliferation and differentiation. Stem Cell Rev 5, 231–246 (2009). 19562527 10.1007/s12015-009-9080-2 PMC10467564

[b17] Ozkucur N., Perike S., Sharma P. & Funk R. H. Persistent directional cell migration requires ion transport proteins as direction sensors and membrane potential differences in order to maintain directedness. BMC Cell Biol 12, 4 (2011). 21255452 10.1186/1471-2121-12-4 PMC3042415

[b18] Owens D. F., Boyce L. H., Davis M. B. & Kriegstein A. R. Excitatory GABA responses in embryonic and neonatal cortical slices demonstrated by gramicidin perforated-patch recordings and calcium imaging. J Neurosci 16, 6414–6423 (1996). 8815920 10.1523/JNEUROSCI.16-20-06414.1996 PMC6578913

[b19] Ben-Ari Y. Excitatory actions of gaba during development: the nature of the nurture. Nat Rev Neurosci 3, 728–739 (2002). 12209121 10.1038/nrn920

[b20] Shan Q., Haddrill J. L. & Lynch J. W. Ivermectin, an unconventional agonist of the glycine receptor chloride channel. J Biol Chem 276, 12556–12564 (2001). 11278873 10.1074/jbc.M011264200

[b21] Mandal S., Lindgren A. G., Srivastava A. S., Clark A. T. & Banerjee U. Mitochondrial function controls proliferation and early differentiation potential of embryonic stem cells. Stem Cells 29, 486–495 (2011). 21425411 10.1002/stem.590 PMC4374603

[b22] Kidokoro Y. Developmental changes of membrane electrical properties in a rat skeletal muscle cell line. J Physiol 244, 129–143 (1975). 1168256 10.1113/jphysiol.1975.sp010787 PMC1330748

[b23] Stambrook P. J., Sachs H. G. & Ebert J. D. The effect of potassium on the cell membrane potential and the passage of synchronized cells through the cell cycle. J Cell Physiol 85, 283–291 (1975). 1168203 10.1002/jcp.1040850215

[b24] Binggeli R. & Weinstein R. C. Membrane potentials and sodium channels: hypotheses for growth regulation and cancer formation based on changes in sodium channels and gap junctions. J Theor Biol 123, 377–401 (1986). 2443763 10.1016/s0022-5193(86)80209-0

[b25] Zundorf G., Kahlert S. & Reiser G. Gap-junction blocker carbenoxolone differentially enhances NMDA-induced cell death in hippocampal neurons and astrocytes in co-culture. J Neurochem 102, 508–521 (2007). 17403140 10.1111/j.1471-4159.2007.04509.x

[b26] Fanarraga M. L., Avila J. & Zabala J. C. Expression of unphosphorylated class III beta-tubulin isotype in neuroepithelial cells demonstrates neuroblast commitment and differentiation. Eur J Neurosci 11, 516–527 (1999). 10051752

[b27] Chu Y., Hughes S. & Chan-Ling T. Differentiation and migration of astrocyte precursor cells and astrocytes in human fetal retina: relevance to optic nerve coloboma. FASEB J 15, 2013–2015 (2001). 11511521 10.1096/fj.00-0868fje

[b28] Noctor S. C. . Dividing precursor cells of the embryonic cortical ventricular zone have morphological and molecular characteristics of radial glia. J Neurosci 22, 3161–3173 (2002). 11943818 10.1523/JNEUROSCI.22-08-03161.2002 PMC6757532

[b29] Guo Z. . Early postnatal GFAP-expressing cells produce multilineage progeny in cerebrum and astrocytes in cerebellum of adult mice. Brain Res 1532, 14–20 (2013). 23939222 10.1016/j.brainres.2013.08.003

[b30] Sensenbrenner M., Lucas M. & Deloulme J. C. Expression of two neuronal markers, growth-associated protein 43 and neuron-specific enolase, in rat glial cells. J Mol Med (Berl) 75, 653–663 (1997). 9351704 10.1007/s001090050149

[b31] Ben-Ari Y. & Spitzer N. C. Phenotypic checkpoints regulate neuronal development. Trends Neurosci 33, 485–492 (2010). 20864191 10.1016/j.tins.2010.08.005 PMC2963711

[b32] Xu X. . Mitochondrial regulation in pluripotent stem cells. Cell Metab 18, 325–332 (2013). 23850316 10.1016/j.cmet.2013.06.005

[b33] Bonnet S. . A mitochondria-K+ channel axis is suppressed in cancer and its normalization promotes apoptosis and inhibits cancer growth. Cancer Cell 11, 37–51 (2007). 17222789 10.1016/j.ccr.2006.10.020

[b34] Sutor B. & Hagerty T. Involvement of gap junctions in the development of the neocortex. Biochim Biophys Acta 1719, 59–68 (2005). 16225838 10.1016/j.bbamem.2005.09.005

[b35] Zhou F. M. & Hablitz J. J. Postnatal development of membrane properties of layer I neurons in rat neocortex. J Neurosci 16, 1131–1139 (1996). 8558242 10.1523/JNEUROSCI.16-03-01131.1996 PMC6578792

[b36] Spigelman I., Zhang L. & Carlen P. L. Patch-clamp study of postnatal development of CA1 neurons in rat hippocampal slices: membrane excitability and K+ currents. J Neurophysiol 68, 55–69 (1992). 1517828 10.1152/jn.1992.68.1.55

[b37] Hedrick T. & Waters J. Effect of temperature on spiking patterns of neocortical layer 2/3 and layer 6 pyramidal neurons. Front Neural Circuits 6, 28 (2012). 22593736 10.3389/fncir.2012.00028 PMC3350897

[b38] Pao G. M. . Role of BRCA1 in brain development. Proc Natl Acad Sci USA 111, E1240–1248 (2014). 24639535 10.1073/pnas.1400783111 PMC3977248

[b39] Faratian D., Munro A., Twelves C. & Bartlett J. M. Membranous and cytoplasmic staining of Ki67 is associated with HER2 and ER status in invasive breast carcinoma. Histopathology 54, 254–257 (2009). 19207951 10.1111/j.1365-2559.2008.03191.x

[b40] Moll U. M., Riou G. & Levine A. J. Two distinct mechanisms alter p53 in breast cancer: mutation and nuclear exclusion. Proc Natl Acad Sci USA 89, 7262–7266 (1992). 1353891 10.1073/pnas.89.15.7262 PMC49686

[b41] Kato Y. . Acidic extracellular microenvironment and cancer. Cancer Cell Int 13, 89 (2013). 24004445 10.1186/1475-2867-13-89 PMC3849184

[b42] He S., Li Z., Ge S., Yu Y. C. & Shi S. H. Inside-Out Radial Migration Facilitates Lineage-Dependent Neocortical Microcircuit Assembly. Neuron 86, 1159–1166 (2015). 26050035 10.1016/j.neuron.2015.05.002 PMC4458701

[b43] Palm G., Knoblauch A., Hauser F. & Schuz A. Cell assemblies in the cerebral cortex. Biol Cybern 108, 559–572 (2014). 24692024 10.1007/s00422-014-0596-4

[b44] Sanai N. . Corridors of migrating neurons in the human brain and their decline during infancy. Nature 478, 382–386 (2011). 21964341 10.1038/nature10487 PMC3197903

[b45] Bronner-Fraser M. & Fraser S. E. Differentiation of the vertebrate neural tube. Curr Opin Cell Biol 9, 885–891 (1997). 9425355 10.1016/s0955-0674(97)80092-0

[b46] Engler A. J., Sen S., Sweeney H. L. & Discher D. E. Matrix elasticity directs stem cell lineage specification. Cell 126, 677–689 (2006). 16923388 10.1016/j.cell.2006.06.044

[b47] Klein C. . Enhanced adult neurogenesis increases brain stiffness: *in vivo* magnetic resonance elastography in a mouse model of dopamine depletion. Plos One 9, e92582 (2014). 24667730 10.1371/journal.pone.0092582 PMC3965445

[b48] Iwashita M., Kataoka N., Toida K. & Kosodo Y. Systematic profiling of spatiotemporal tissue and cellular stiffness in the developing brain. Development 141, 3793–3798 (2014). 25249464 10.1242/dev.109637

[b49] Ozkucur N. . Membrane potential depolarization causes alterations in neuron arrangement and connectivity in cocultures. Brain Behav 5, 24–38 (2015). 25722947 10.1002/brb3.295 PMC4321392

[b50] Cao L. . Endogenous electric currents might guide rostral migration of neuroblasts. EMBO Rep 14, 184–190 (2013). 23328740 10.1038/embor.2012.215 PMC3596136

[b51] Pai V. P. . Endogenous gradients of resting potential instructively pattern embryonic neural tissue via Notch signaling and regulation of proliferation. J Neurosci 35, 4366–4385 (2015). 25762681 10.1523/JNEUROSCI.1877-14.2015 PMC4355204

[b52] Brewer G. J., Torricelli J. R., Evege E. K. & Price P. J. Optimized survival of hippocampal neurons in B27-supplemented Neurobasal, a new serum-free medium combination. J Neurosci Res 35, 567–576 (1993). 8377226 10.1002/jnr.490350513

[b53] Lesuisse C. & Martin L. J. Long-term culture of mouse cortical neurons as a model for neuronal development, aging, and death. J Neurobiol 51, 9–23 (2002). 11920724 10.1002/neu.10037

[b54] Rockwood D. N. . Materials fabrication from Bombyx mori silk fibroin. Nat Protoc 6, 1612–1631 (2011). 21959241 10.1038/nprot.2011.379 PMC3808976

[b55] Nazarov R., Jin H. J. & Kaplan D. L. Porous 3-D scaffolds from regenerated silk fibroin. Biomacromolecules 5, 718–726 (2004). 15132652 10.1021/bm034327e

[b56] Almeida A., Almeida J., Bolanos J. P. & Moncada S. Different responses of astrocytes and neurons to nitric oxide: the role of glycolytically generated ATP in astrocyte protection. Proc Natl Acad Sci USA 98, 15294–15299 (2001). 11742096 10.1073/pnas.261560998 PMC65023

[b57] Cassina P. . Mitochondrial dysfunction in SOD1G93A-bearing astrocytes promotes motor neuron degeneration: prevention by mitochondrial-targeted antioxidants. J Neurosci 28, 4115–4122 (2008). 18417691 10.1523/JNEUROSCI.5308-07.2008 PMC3844766

