# Supplementary material for: A biofidelic 3D culture model to study the development of brain cellular systems
Source: Sci Rep. 2016 Apr 26;6:24953. doi: 10.1038/srep24953 (PMC4844952; doi:10.1038/srep24953)
Supplement: Supplementary Information [file srep24953-s1.pdf]

A biofidelic 3D culture model to study the development of brain cellular systems

Ren M[1], Du C[1], Herrero Acero E[1,3], Tang-Schomer M[1,4], Özkucur N[1,2,*]

[1]Tufts University, Department of Biomedical Engineering, 4 Colby St., Medford, Massachusetts 02155, USA; [2]Department of Biology, Tufts University, 200 Boston Avenue, Suite 4600, Medford, Massachusetts 02155, USA; [3]Austrian Centre of Industrial Biotechnology Konrad Lorenz Strasse 20, A-3430, Tulln, Austria; [4]Department of Pediatrics, Connecticut Children's Medical Center & UConn Health, 263 Farmington Avenue, Farmington, Connecticut 06030, USA.

*nurdan.oezkucur@tufts.edu

Supplementary Information

Supplementary Figure S1:

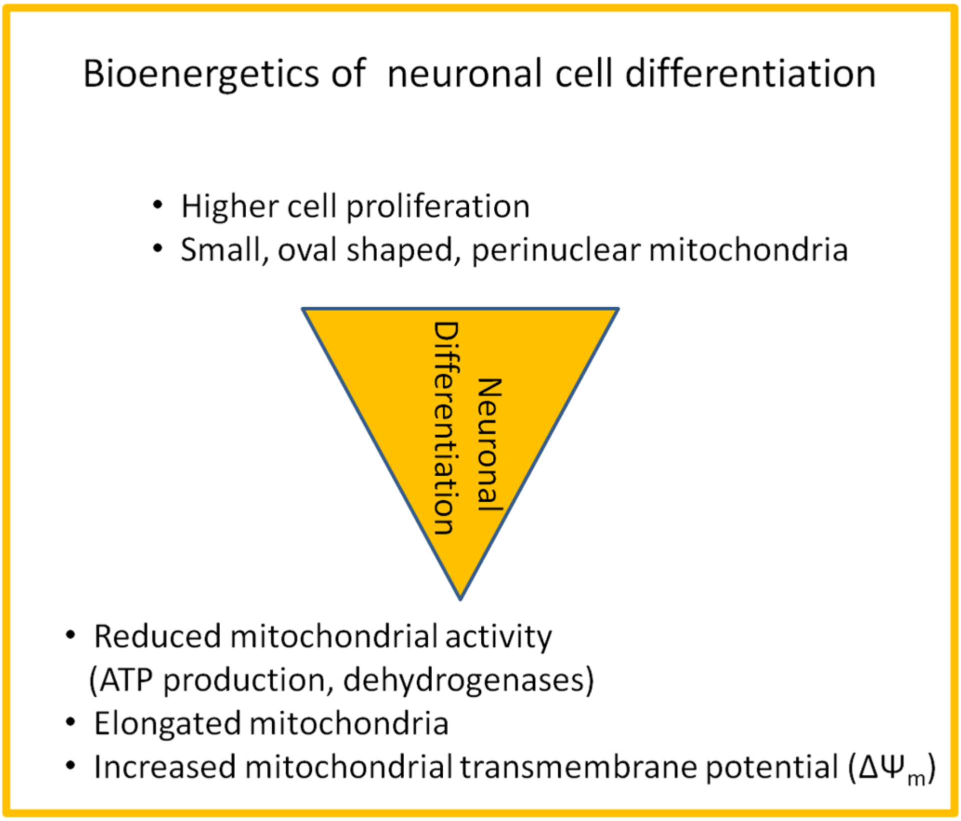

Figure S1: Summary of the mitochondria-related changes that occur during neuronal cell differentiation.

Supplementary Data S2:

V_{mem}-induced changes in $\Delta\Psi_m$ can, in combination with organelle shape, reflect different stages of cellular differentiation, including tumor initiation

It is widely accepted that tumor cells exhibit a characteristic $\Delta\Psi_m$ hyperpolarization, and several studies have demonstrated that tumorigenic properties can be stopped by arresting the G1 phase of the cell cycle when the $\Delta\Psi_m$ is depolarized [1]. Moreover, as described by Otto Warburg in 1924 [2], elevated glycolysis is a central hallmark of all tumor types (including brain tumors) that has been used in clinical tumor diagnosis for decades. In addition, $\Delta\Psi_m$ depolarization is a hallmark of apoptosis in many cell types in both adults and during development. However, this finding excludes neurons [3]. In neural tissues, where and when $\Delta\Psi_m$ depolarization occurs has more to do with reprogramming (e.g., acquiring stem cell metabolism) than cancer or developmental apoptosis. The differentiation of neural progenitor/stem cells from tumorigenic cells is dependent on differences in their glycolytic mechanisms. This differentiation is highly relevant to the validation of healthy stem/progenitor cell features in a given population because such evaluations can be applied both *in vivo* (e.g., in early monitoring and diagnosis in the developing brain) and *in vitro* (e.g., when reprogramming cells for therapy). This finding also provides insight into the cell type-specific developmental pathways that result in different tumor phenotypes. Importantly, in Ivm-treated cells, the presence of astrocytes caused the $\Delta\Psi_m$ to be consistently depolarized (lower JC1 ratio), indicating that cells in the co-cultures gained stem cell-like features rather than tumorigenic features [4-6].

In 2D cultures, elongated mitochondria were observed in control cells, similar to what occurs in differentiated cells, whereas the cells displaying a depolarized V_{mem} exhibited smaller, punctate-like mitochondria, which are indicative of pluripotent cells [7-10]. The activity-dependent shape of the mitochondria is a recently discovered characteristic of stem cells [6]. In 2D cultures of E18 rat primary cortical astrocytes, diverse cell types with different morphologies and different Ψ_m levels (green monomers vs red aggregates following labeling with JC1) were observed in the control cultures after 4 wks (Fig. S2Aa-c). Notably, after 4 wks of treatment with Ivm, the astrocyte cultures consisted of cells with different morphologies that resembled newborn neurons/progenitors (Fig. S2 Ad), postmitotic neurons (Fig. S2Ae),

immature astrocytes (red, punctate mitochondria in Fig. S2Af) and oligodendrocytes. These cells were observed to be sitting along the nerve cells (the large, red cells in Fig. S2Af). In contrast, neither the co-cultures nor the neuron cultures grown with or without Ivm showed diverse cell morphologies when they were stained with JC1 (Fig. S2Ba-e, Fig. S2Ca, b). However, the mitochondria of the Ivm-treated cells in the co-cultures differed in morphology and predominantly showed shorter and more punctate shapes (close-up view in Fig. S2Be) than were observe in the control cells (see the close-up view in Fig. S2Ba).

Enhanced $\Delta\Psi_m$ is associated with the process of neurite outgrowth [9] and is also characteristic of tumor cell physiology [11, 12]. We therefore examined whether $\Delta\Psi_m$ was also associated with the V_{mem} of cells during differentiation in rat primary cortical neurons *in vitro*. Homotypic astrocyte cultures presented a decrease in $\Delta\Psi_m$ after 1 wk and an increase after 2 wks (Fig. S2D). However, the results indicated that their V_{mem} changed only slightly after 2 wks and 4 wks *in vitro*. When co-cultured with astrocytes, the neurons showed a reduced $\Delta\Psi_m$ at all time points following exposure to Ivm. This effect was eliminated at all time points when the cells were cultured in the presence of the gap junction (GJ) blockers lindane and octanol (Fig. S2E). When grown in homotypic cultures, E18 rat primary cortical neurons displayed an increased $\Delta\Psi_m$ after 1 wk and a decreased $\Delta\Psi_m$ after 2 wks and 3 wks *in vitro*. After 4 wks, no change in $\Delta\Psi_m$ was detected (Fig. S2F).

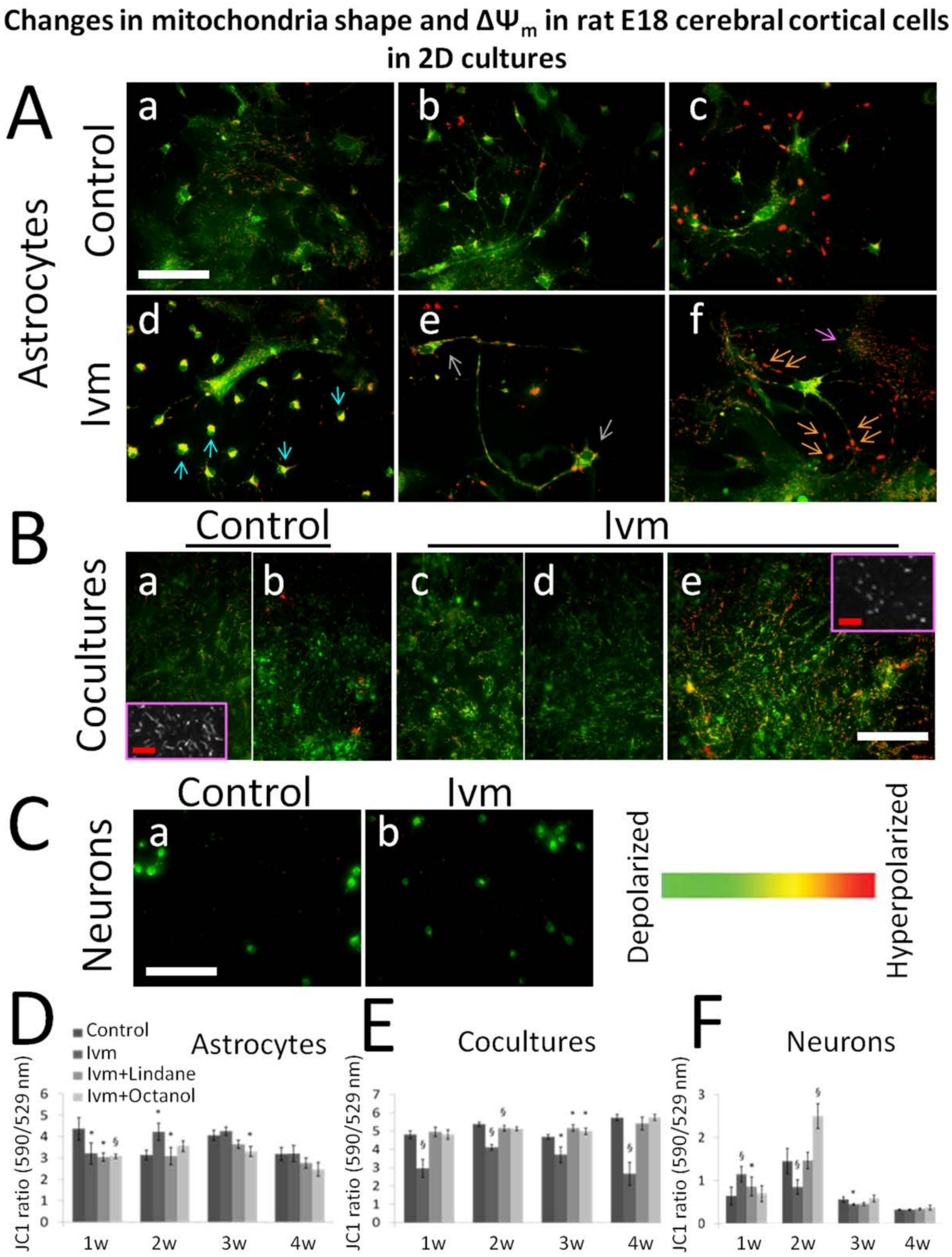

Figure S2: Changing the V_{mem} of cells via gap junctions can change the direction of metabolic progression during cortical neural differentiation in E18 rat primary cultures. Differences in overall cell morphology and $\Delta\Psi_m$ were assessed using JC1 dye in control versus 1 μM Ivm-treated cells. The E18 rat primary cortical astrocytes displayed diverse cell type morphologies and $\Delta\Psi_m$ that were different from those observed in the control cultures after 4 wks (Aa-c). After 4 wks of Ivm treatment, the astrocyte cultures appeared to contain newborn neurons/progenitors (blue arrows in Ad), postmitotic neurons (grey arrows in Ae), immature astrocytes (red, punctate mitochondria in Af, purple arrow) and oligodendrocytes. These cells were observed to be sitting along the nerve cells (the large, red cells in Af, orange arrows). Co-cultures and neuron cultures grown with or without Ivm did not show a diversity of cell morphologies when they were stained with JC1 (Ba-e, Ca, b). As the images of green JC1

monomers indicate, immature (shorter) mitochondria were observed in the cells treated with 1 µM Ivm (Be, close-up view in purple window). These results were in contrast to the results in the control cells, which had elongated mitochondria (Ba, close-up view in purple window), suggesting a mature state. Bar: 50 µm (5 µm in close up views). $\Delta\Psi_m$ was measured as a marker of metabolic maturation in the control and 1 µM Ivm-treated astrocytes (D), neurons (E) and co-cultures (F). Measurements were also taken in the presence of the gap junction blockers lindane and octanol to demonstrate that the effects of astrocytes that are located next to the neurons are mediated by gap junction connections between these cell types. Reversing the cell membrane potential relatively hyperpolarized the $\Delta\Psi_m$ in neuron-astrocyte co-cultures. This effect was ruled out in the presence of gap junction blockers in all 1-, 2-, 3- and 4 wks cocultures (F). *$p \leq 0.05$, §$p \leq 0.01$* versus the control. N = 16 wells in 96-well plates from three separate cultures.

Supplementary Figure S3:

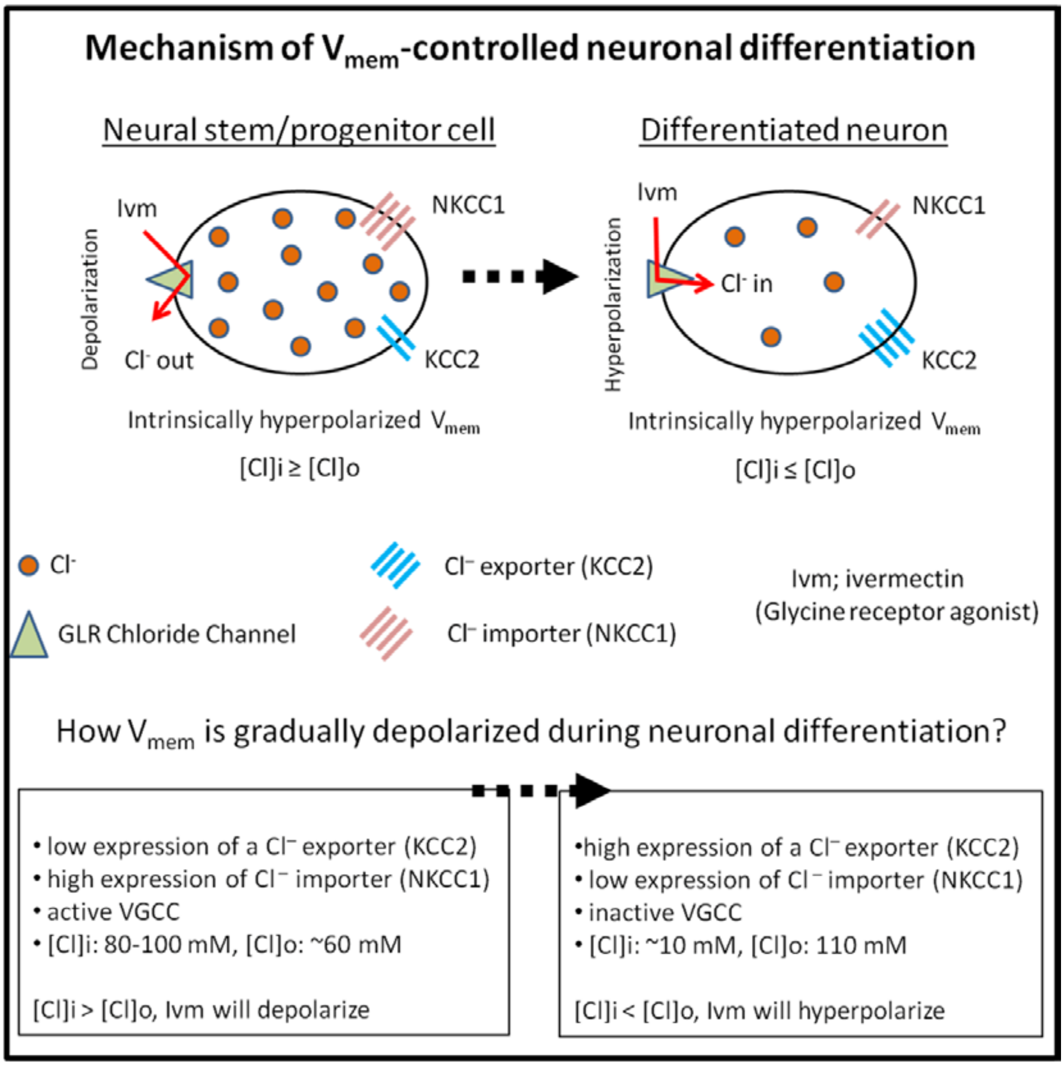

Figure S3: Summary diagram demonstrating the mechanism of V_{mem}-controlled neuronal differentiation.

Supplementary Figure S4:

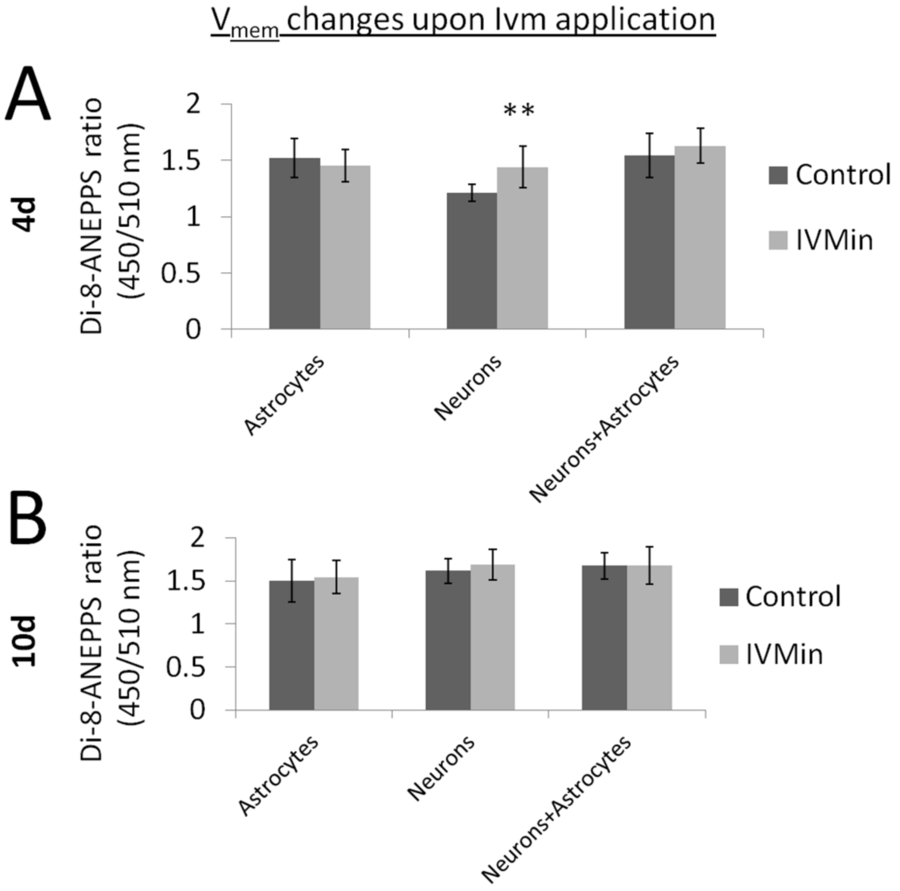

Figure S4: Four-day-old cultures of neurons exhibited significantly depolarized (%25) V_{mem} following treatment with Ivm, whereas V_{mem} was unchanged in astrocyte-only cultures and co-cultures (Fig. S4A). This result indicates that when Ivm treatment was applied within a specific time window (0-4 days *in vitro*) in our study, the neuron population was selectively targeted. When Ivm was added to ten-day-old cultures, Ivm did not change the V_{mem} of the cells (Fig. S4B).

Supplementary Figure S5:

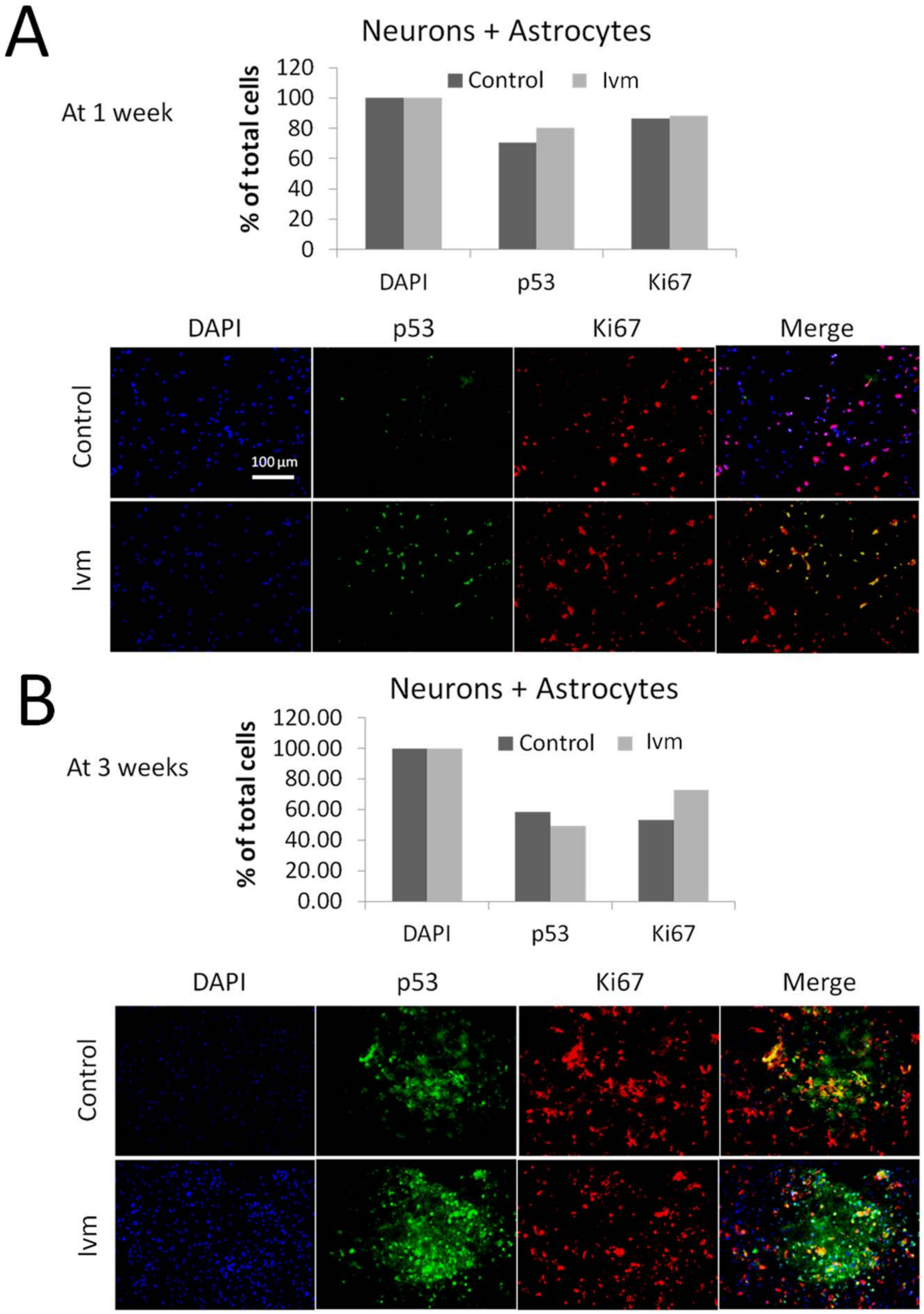

Figure S5: Intracellular production of the tumor marker p53 and the cell proliferation marker Ki67 in E18 rat primary cortical 2D cocultures during V_{mem} manipulation-induced reprogramming. (A) Images

showing cells after 1 wk in culture that were stained with p53 (Alexa Fluor 488) and Ki67 (Alexa Fluor 568). The plot shows that the percentage of p53-positive cells increases, while there was no change in the number of proliferating cells (Ki67+) after 1 wk of treatment with Ivm. (B) Images showing cells grown in culture for 3 wks and then stained for p53 (Alexa Fluor 488) and Ki67 (Alexa Fluor 568). The plot shows that the percentage of p53-positive cells decreases despite the increase in the number of proliferating cells (Ki67+) after 3 wks of treatment with Ivm. N=2 each with ≥300 cells.

Supplementary Data S6:

Dynamic drug adsorption on silk films via QCM

Drug adsorption on silk films was monitored using a quartz crystal microbalance with dissipation (QCM-D) by Q-Sense AB (Gothenburg, Sweden).

The quartz sensors that were used possessed a gold plate electrode and silica on the active surface. The sensors were span-coated with a 1% silk solution and boiled for 30 minutes in water using the following program: from 0 to 500 rpm in 5 seconds, hold for 10 seconds, up to 500 rpm in 10 seconds, hold at this speed for 60 seconds, and down to 0 rpm in 5 seconds. The spin coater that was used was a WS-400 W-6NPP/LITE (Laurell Technologies, USA). After spin coating, the sensors were post-treated using water annealing at 25°C for 12 hours according to a procedure that was previously described by Hu X. et al [1]. This post-treatment results in films with a crystallinity of 30% [1].

Once the silk film was deposited on the sensors, deionized water was flown into the measurement chamber at a rate of 100 µl/min until a stable baseline was reached, in terms of frequency and dissipation. Subsequently, a solution containing 0.01% dimethylsulfoxide (DMSO) was flushed into the measurement chamber at the same flow rate until a stable baseline was reached. Solutions containing 0.01% DMSO and 0.01% of the drug (from a 10 mM stock solution in DMSO) in deionized water were sequentially pumped into the measuring chamber to monitor adsorption.

QCM analysis of the dynamic adsorption of Ivm on 30% crystalline 30 mb silk films showed that there was no significant or measurable adsorption of either DMSO or Ivm on the silk films (Figure S6).

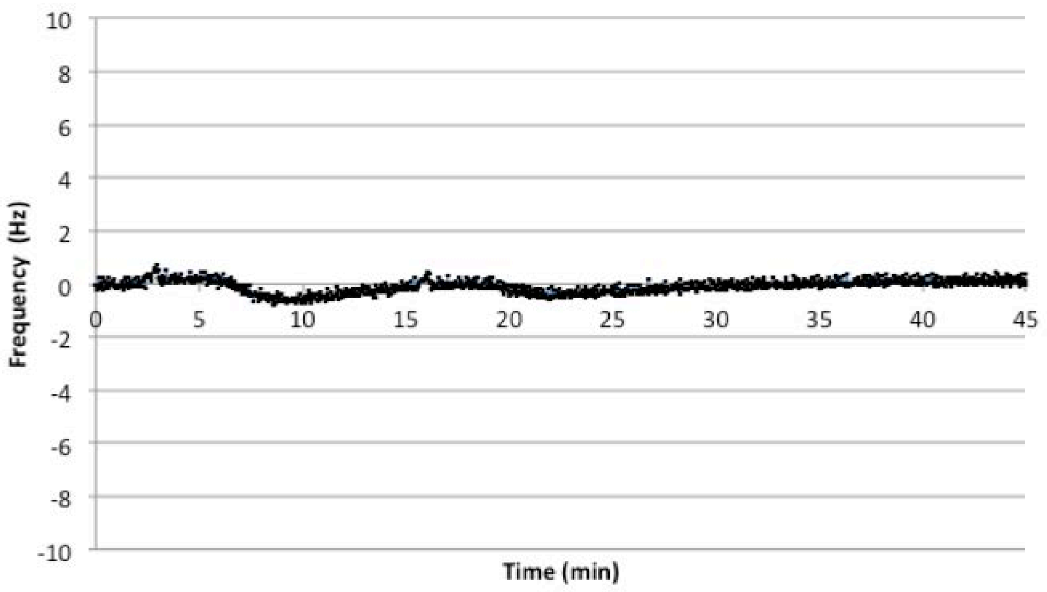

Figure S6: QCM analysis (7th overtone frequency changes) of 0.01% DMSO or Ivm in 0.01% DMSO adsorption on 30 mb 30 % crystalline silk films.

The introduction of the 0.01% DMSO solution to the measurement chamber after 6 minutes barely decreased the frequency of 0.8 Hz before there was a quick recovery to the initial frequency level at baseline. The subsequent introduction, after 20 minutes, of the Ivm in 0.01% DMSO solution achieved only an insignificant frequency decrease of 0.5 Hz. Similarly, the continuous flow of the drug solution induced a stabilization of the baseline around the initial base level. The changes in the dissipation during the whole experiment were less than 0.1 Hz. Both the frequency and the dissipation were marginally changed, indicating a non-significant level of Ivm adsorption on the silk films.

References

1. Bonnet, S. et al. A mitochondria-K+ channel axis is suppressed in cancer and its normalization promotes apoptosis and inhibits cancer growth. *Cancer Cell* **11**, 37-51 (2007).
2. Warburg, O. On the origin of cancer cells. *Science* **123**, 309-314 (1956).
3. Krohn, A.J., Wahlbrink, T. & Prehn, J.H. Mitochondrial depolarization is not required for neuronal apoptosis. *J Neurosci* **19**, 7394-7404 (1999).
4. Suhr, S.T. et al. Mitochondrial rejuvenation after induced pluripotency. *PLoS One* **5**, e14095 (2010).
5. Schieke, S.M. et al. Mitochondrial metabolism modulates differentiation and teratoma formation capacity in mouse embryonic stem cells. *J Biol Chem* **283**, 28506-28512 (2008).

6. Mandal, S., Lindgren, A.G., Srivastava, A.S., Clark, A.T. & Banerjee, U. Mitochondrial function controls proliferation and early differentiation potential of embryonic stem cells. *Stem Cells* **29**, 486-495 (2011).
7. Mitra, K. Mitochondrial fission-fusion as an emerging key regulator of cell proliferation and differentiation. *Bioessays* **35**, 955-964 (2013).
8. Kasahara, A., Cipolat, S., Chen, Y., Dorn, G.W., 2nd & Scorrano, L. Mitochondrial fusion directs cardiomyocyte differentiation via calcineurin and Notch signaling. *Science* **342**, 734-737 (2013).
9. Voccoli, V. & Colombaioni, L. Mitochondrial remodeling in differentiating neuroblasts. *Brain Res* **1252**, 15-29 (2009).
10. Xu, X. et al. Mitochondrial regulation in pluripotent stem cells. *Cell Metab* **18**, 325-332 (2013).
11. Preston, T.J., Abadi, A., Wilson, L. & Singh, G. Mitochondrial contributions to cancer cell physiology: potential for drug development. *Adv Drug Deliv Rev* **49**, 45-61 (2001).
12. Hockenbery, D.M. Targeting mitochondria for cancer therapy. *Environ Mol Mutagen* **51**, 476-489 (2010).
